# Multifunctional bending magnet beamline with a capillary optic for X-ray fluorescence studies of metals in tissue sections

**DOI:** 10.1107/S1600577526000925

**Published:** 2026-02-19

**Authors:** Benjamin Roter, Andrew M. Crawford, Qiaoling Jin, Arthur T. Glowacki, Barry Lai, Fabricio S. Marin, Evan Maxey, Xianbo Shi, Valeria C. Culotta, Asia S. Wildeman, Naisargi K. Patel, Thomas V. O’Halloran, Chris Jacobsen

**Affiliations:** a Applied Physics Program Northwestern University Evanston IL60208 USA; b Department of Microbiology, Genetics and Immunology Michigan State University East Lansing USA; c Department of Chemistry Michigan State University East Lansing MI48824 USA; d Department of Physics and Astronomy Northwestern University Evanston IL60208 USA; e Chemistry of Life Processes Institute Northwestern University Evanston IL60208 USA; f X-ray Science Division, Advanced Photon Source Argonne National Laboratory Lemont IL60439 USA; g Department of Biochemistry and Molecular Biology, Bloomberg School of Public Health Johns Hopkins University Baltimore MD21205 USA; h Elemental Health Institute Michigan State University East Lansing MI48824 USA; Brazilian Synchrotron Light Laboratory, Brazil

**Keywords:** X-ray microscopy, X-ray fluorescence, capillary optic, elemental analysis

## Abstract

Scanning fluorescence X-ray microscopy lets one non-destructively and quantitatively map the distribution of most biologically important metals in cells and tissues. For studies on large-scale tissues and organs, a spatial resolution of several micrometres is often sufficient; in this case, bending magnets at synchrotron light sources provide abundant X-ray flux. We describe here the use of bending magnet beamline 8-BM-B at the Advanced Photon Source with two distinct microscopy stations: a pre-existing one with Kirkpatrick–Baez (KB) mirror optics for slightly higher throughput and the ability to accommodate samples tens of centimetres across, and a new prototype station with an axially symmetric, single-bounce, capillary optic with slightly less flux, but finer resolution at similar fluence per time. The KB station provides δ_res_ = 10.5 µm spatial resolution at a per-pixel exposure time of *t*_dwell_ = 100 ms and a fluence per time of 5.8 × 10^7^ photons µm^−2^ s^−1^, while the prototype capillary station provides δ_res_ = 6.5 µm at *t*_dwell_ = 50 ms and a fluence per time of 5.6 × 10^7^ photons µm^−2^ s^−1^. We used image power spectral density to estimate the achieved spatial resolution δ_res_ from individually acquired images, with δ_res_ depending on the optic, the fluorescence signal strength of the sample being imaged, and the method used to process raw fluorescence spectral data.

## Abstract 2

We describe a bending magnet endstation for X-ray fluorescence studies of metal distributions in millimetre-sized biological specimens. An existing setup using Kirkpatrick–Baez mirrors with 10.5 µm spatial resolution was supplemented with a second scanning setup with a capillary optic with 6.5 µm resolution.

## 1. Introduction

Quantitative methods for mapping elemental distributions are essential for addressing a wide range of problems in the life sciences (McRae *et al.*, 2009 ▸; Zee *et al.*, 2022 ▸). To understand the function of metals in thin sections from tissues and organs, quantitative imaging at a spatial resolution of several micrometres can provide information on cell-to-cell metal variations, while a field of view of several millimetres allows one to image large, representative regions of organs from small animals. For elements with atomic numbers above about *Z* = 14, scanning fluorescence X-ray microscopy (SFXM) offers a very useful combination of high sensitivity with relatively low beam damage (Kirz, 1980 ▸; Sparks, 1980 ▸; Pushie *et al.*, 2022 ▸). In this approach, a small specimen is raster-scanned through an X-ray beam spot, and an energy-dispersive detector is used to record the emission at characteristic X-ray fluorescence lines (Horowitz & Howell, 1972 ▸; Sparks, 1980 ▸; Jones *et al.*, 1984 ▸). The recorded signal includes background from elastic and Compton X-ray scattering, as well as other factors such as incomplete charge collection in energy-dispersive detectors (Van Grieken & Markowicz, 2002 ▸). A variety of analysis software packages can be used to separate signal from background, delivering quantitative measures of elemental concentration (Ryan, 2000 ▸; Vogt, 2003 ▸; Solé *et al.*, 2007 ▸; Schoonjans *et al.*, 2012 ▸; Schoonjans *et al.*, 2013 ▸; Crawford *et al.*, 2019 ▸).

Obtaining high resolution in SFXM requires an X-ray source providing significant flux within a small area with narrow solid angle; that is, a source with high spectral brightness (Jacobsen, 2020 ▸). Emission from such a source can then be demagnified by an optic to a small spot through which the sample is scanned. For nanoscale imaging within single cells, undulator sources in straight sections of low-emittance storage rings provide the highest possible brightness (Eriksson *et al.*, 2014 ▸) outside of free-electron lasers. When evaluating overall elemental distributions in biological tissues or organs, micrometre-scale imaging can provide megapixel images of samples that are millimetres in size. In this case, the somewhat larger source size and divergence of bending magnet (dipole) sources at synchrotron light sources can provide quite satisfactory performance, often with easier access for imaging many samples.

We describe here the use of bending magnet beamline 8-BM-B at the Advanced Photon Source (APS) at Argonne National Laboratory, USA. This beamline has a pre-existing SFXM station that uses a Kirkpatrick–Baez (KB) mirror (Kirkpatrick & Baez, 1948 ▸) originally designed for use at a separate synchrotron beamline. As part of a project for Quantitative Elemental Mapping for the Life Sciences (QE-MAP), which is an NIH-supported Biomedical Technology Research Resource based at Michigan State University, we have added a second prototype SFXM station just downstream. It is equipped with a capillary focusing optic for higher spatial resolution, and a fluorescence detector with greater signal collection capability. These two stations will be described in more detail in Section 1.2.

### 1.1. Example: metals in a mouse kidney

**Figure 1:**
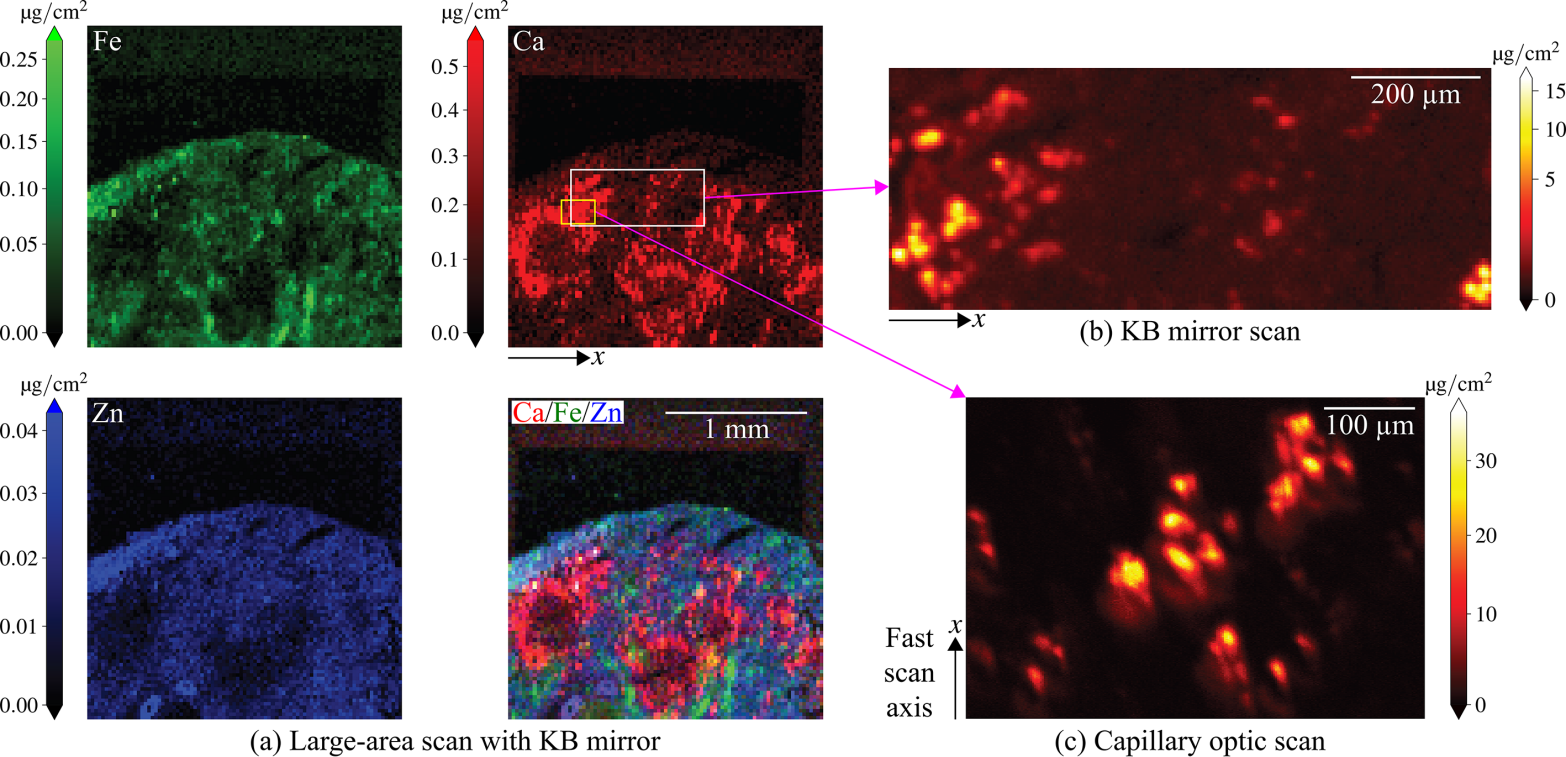
Scanning fluorescence X-ray microscopy (SFXM) images elemental distributions at different length scales. Shown here are trace essential metals inside a sample of a mouse kidney partial section infected with *Candida albicans*, with Ca being strongly elevated at sites of infection. We obtained the images in (*a*) using the KB mirror scanning station at 8-BM with a 25 µm step size at *t*_dwell_ = 50 ms. These metals are also displayed in a color composite map, allowing one to more easily see how the elements are differentially distributed in the specimen. The images in (*b*) and (*c*) correspond to regions we selected from (*a*) using the KB mirror and capillary optics, respectively. In (*b*), we used a Δ_*x*_ = Δ_*y*_ = 5 µm step size at *t*_dwell_ = 100 ms. In (*c*), we used step sizes of Δ_*x*_ = 1 µm and Δ_*y*_ = 2 µm in *y*. The spatial resolution improved from 10.5 µm to 6.3 µm when transitioning from the KB optic to the capillary (as described in Section 2.2). The large-scanning area SFXM capabilities of 8-BM described in this work provide an important complement to nanoscale imaging capabilities from other beamlines at the Advanced Photon Source at Argonne National Laboratory.

As an example of SFXM capabilities provided at beamline 8-BM-B, Fig. 1 ▸ shows the distribution of three important metals in a mouse kidney partial section. This particular mouse was infected with *Candida albicans* clinical isolate SC5314 via tail vein injections as part of a larger study aimed at understanding how mammalian hosts attempt to starve invading pathogens of essential nutrient metals such as Fe, Cu, Zn, and Mn (Wildeman *et al.*, 2023 ▸). At 72 h post-infection, the kidney was extracted, embedded in an optimum cutting temperature (OCT) compound (Tissue-Tek), and frozen in an isopentane bath chilled with liquid N_2_-cooled isopentane. We cut the frozen block into sections of 10 µm thickness while using a cryostat (CM3050S, Leica) that maintained them at −15 to 18°C during the process. The sections were then transferred to Si_3_N_4_ chips (NX5200, Norcada) previously affixed to glass slides by Kapton tape. We stored the sections in airtight slide boxes in a −80°C freezer until retrieval for SFXM analysis. Upon thawing the original airtight slide boxes at room temperature, the chips containing the kidney sections from the glass slides were removed and placed onto a sample mount specifically designed for 8-BM-B (at which point most of the water in the sections evaporated). We scanned those sections at 8-BM-B and used the known areal mass concentrations of calcium, iron, and copper in an AXO 10X thin film standard (RF8-200-S2454, Applied X-ray Optics GmbH), which was scanned separately, to calibrate their metal concentrations. Two-dimensional elemental maps of each section were obtained by performing fluorescence spectrum fitting with *MAPS* (Vogt, 2003 ▸) and *M-BLANK* (Crawford *et al.*, 2019 ▸) software as is described in Section 1.5. In Fig. 1 ▸(*a*), elemental maps of iron, calcium, and zinc are shown as obtained using the KB mirror station with a scan step size of Δ_*x*_ = Δ_*y*_ = 25 µm and a per-pixel exposure time *t*_dwell_ = 50 ms. Also shown in Fig. 1 ▸(*a*) is a three-color composite map of these three elements. The ring-like features in the Ca distribution indicate possible localized infection sites. Figs. 1 ▸(*b*) and 1(*c*) show finer fields of view of localized Ca hotspots at different length scales using (*b*) the KB mirror with Δ_*x*_ = Δ_*y*_ = 5 µm and *t*_dwell_ = 100 ms and (*c*) the capillary optics with Δ_*x*_ = 1 µm, Δ_*y*_ = 2 µm, and *t*_dwell_ = 50 ms. As can be seen, the capillary optic station yields a higher resolution image.

### 1.2. Beamline 8-BM-B at the APS

**Figure 2:**
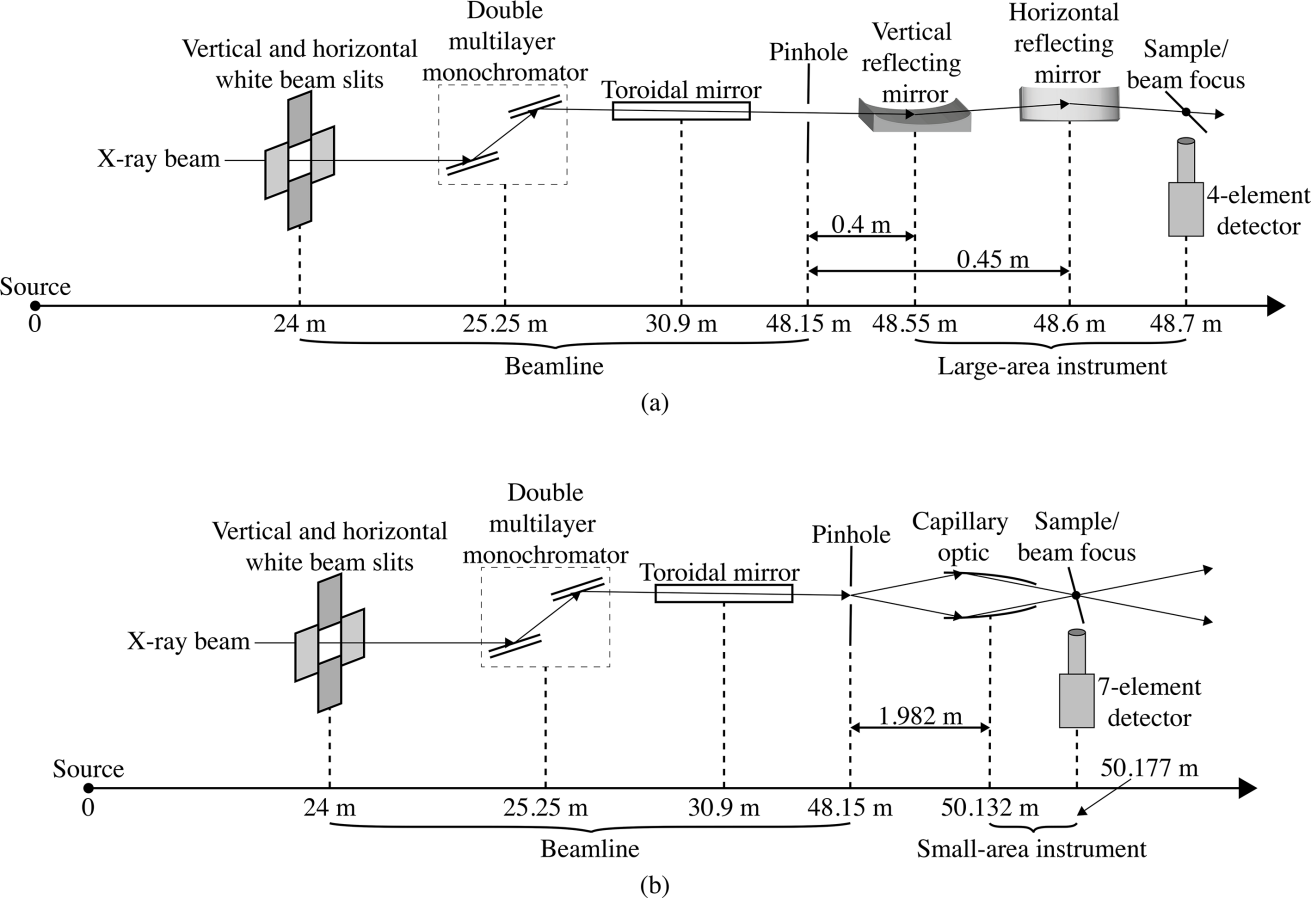
Layout of the 8-BM-B beamline when used with both a pre-existing KB mirror/large-area instrument setup (*a*) and a new, additional capillary/small-area instrument (*b*) located in the experimental enclosure. Both schematics show the location of the double multilayer monochromator (DMM), and the toroidal mirror intended to image the X-ray beam from the storage ring to the exit slit position. The beam size is then set using a pinhole as a beam-defining aperture (BDA). The KB mirror setup is for scanning sample fields up to several centimetres across at about δ_res_ = 10.5 µm spatial resolution, and the capillary optic setup is for δ_res_ = 6.5 µm spatial resolution over smaller fields of view (Section 2.1).

Beamline 8-BM-B at the APS has had an evolving mission, with corresponding changes in its instrumentation. This bending magnet beamline was built in the mid-1990s as a general purpose beamline. Around 2010, it was repurposed for micrometre-scale X-ray microprobe studies. The beamline optical layout at the time of our experiments is shown in Fig. 2 ▸. A double-multilayer monochromator (DMM) located 25.25 m from the source was used to deliver a spectral bandwidth of about Δλ/λ = 0.0109 at a photon energy of 10 keV. While our experiments were carried out at 10 keV photon energy, the beamline can operate over a range 9–18 keV. A set of secondary source apertures were located 48.15 m from the primary source, just inside the experimental enclosure (the ‘hutch’). A vertically deflecting, bendable toroidal mirror (originally designed for a different beamline) was located 30.9 m from the source; it delivered an intermediate focus spot located significantly upstream of the beam-defining aperture (BDA). Within the hutch, the secondary source apertures were located 0.2 m from the upstream end of a 2.4 m long optical table. At the table’s downstream end was an X-ray beam camera consisting of a 1 mm thick cadmium tungstate scintillator (10 mm side length), a 5× microscope objective, and a visible light camera. This camera was used for optic and specimen alignment. The BDA consisted of a 250 µm diameter hole in a 0.25 mm thick platinum plate in front of a 1 mm diameter hole, which was in turn located inside a 12.7 mm thick aluminium plate. At the time of our measurements, the beam profile was roughly 1 mm × 2 mm at the input of the pinhole. There is the potential to enhance beamline performance by upgrading the toroidal mirror, and the source characteristics are being improved by the realization of the APS-Upgrade (APS-U) (Kerby, 2023 ▸).

This beamline already featured a large-scanning-area SFXM setup employing a KB focusing optic mounted in air, with a helium enclosure for the specimen and the detector window. The sample scanning setup used with the KB optic had a large working distance of about 50 mm and a maximum scanning field of view of 250 mm and 150 mm in the horizontal and vertical directions, respectively. To allow for clearance for very large specimens and good X-ray fluorescence signal collection efficiency, the sample was mounted at 45° relative to the incident beam normal. The system was equipped with a four-element silicon drift detector (267-VTX-ME4, Hitachi) with xMAP (XIA) detector readout electronics. Because of the imperfect match between 8-BM-B beamline optics and the KB mirror, the achieved resolution was reduced, as will be seen in Section 2.1. Nevertheless, the KB scan system had proven to be quite successful in studies involving large-area samples (Kirker *et al.*, 2017 ▸; Zelinka *et al.*, 2018 ▸; Copeland-Hardin *et al.*, 2023 ▸; Broda *et al.*, 2024 ▸).

As noted in Section 1, we enhanced the beamline by adding a second SFXM setup just downstream of the KB setup. This second scanning station, which is presently in prototype status rather than in final status, uses an axially symmetric, single-bounce, capillary focusing optic (Sigray, Inc.) designed to deliver a smaller focus with the optical layout of the 8-BM beamline. This glass capillary was pulled under heating conditions by Sigray with the goal of imaging a source located 1.982 m upstream to a focus located 45 mm downstream of the capillary center while maintaining a working distance of 20 mm. The capillary has an entrance diameter of 0.785 mm and an exit diameter of 0.425 mm; with a 30 nm coating of Pt, it is designed to work at photon energies up to 20 keV with no lower photon energy bound. We evaluated the performance of this optic using the 28-ID-B undulator beamline at the APS so as to provide a well collimated X-ray source; with this source, the optic produced a focal spot with a full width at half-maximum (FWHM) size of δ_res,*x*_ = 2.9 µm and δ_res,*y*_ = 3.0 µm at *E* = 13 keV (see Section S1 of the supporting information for more details).

**Figure 3:**
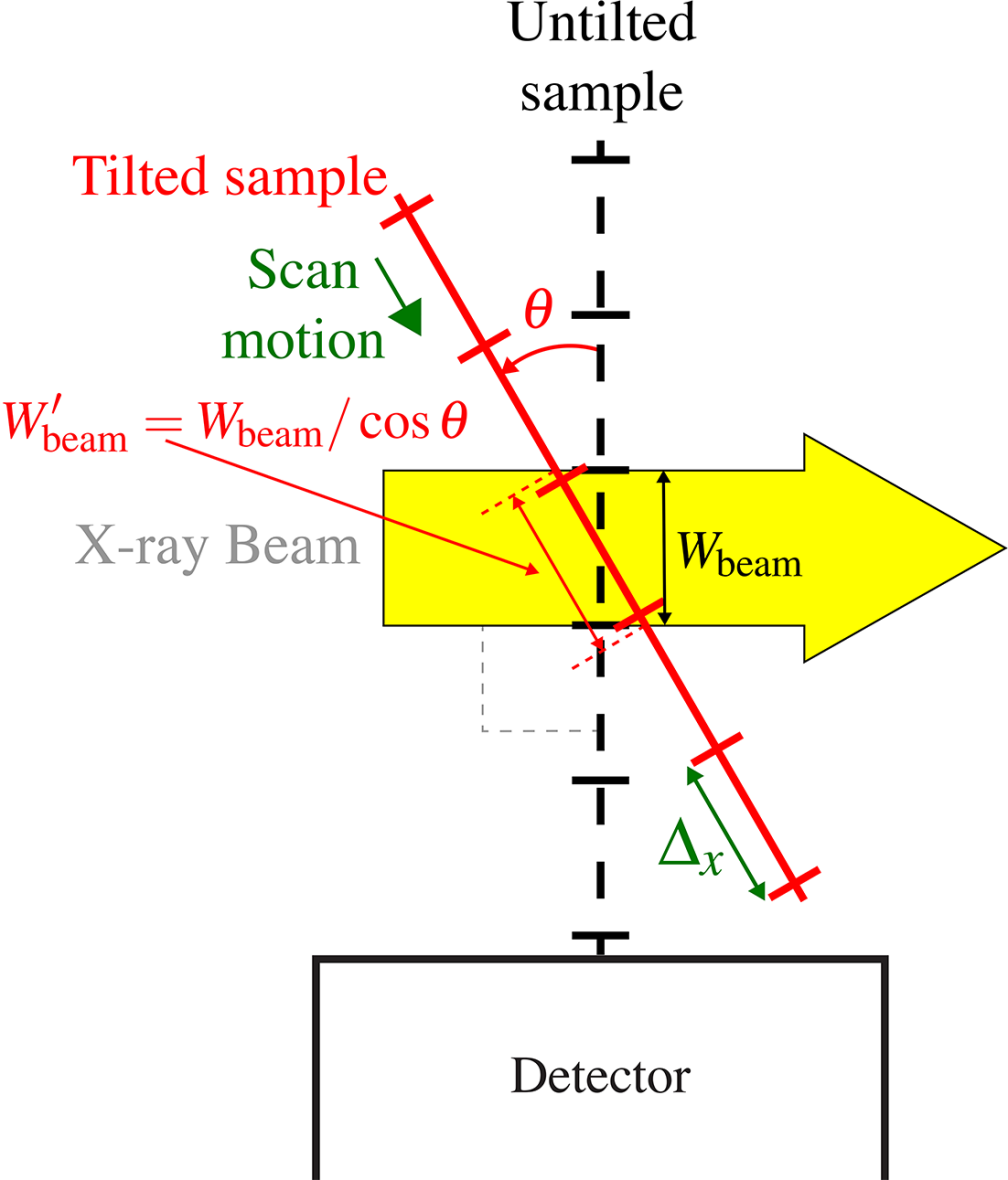
Diagram of how a planar sample and its scanning stage are typically mounted at an angle θ relative to normal incidence for scanning X-ray fluorescence microscopy. This means that the beam width *W*_beam_ as seen by a sample at normal incidence is broadened to 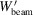 = 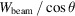 [equation (2)].

The capillary optic and its supplied mount (Sigray, Inc.) is placed on a two-angle tilt stage (8807, New Focus) for angular alignment to the incident beam, with a two-axis Aerotech ATS50 stepper motor stage stack used for optic translation transverse to the beam direction. The alignment of the capillary was adjusted and evaluated by imaging a test pattern. Following the capillary is a micrometre-precision, 50 mm × 50 mm maximum field of view *xyz* scanning stage assembly (*x* and *y* stages: ATS50-50-U-NC, Aerotech; *z* stage: UTM50PP1HL, Newport), with a scanning direction oriented at 15° relative to the incident beam normal (to reduce horizontal beam spreading on the sample; see Fig. 3 ▸). Fluorescence X-ray detection is provided by a seven-element energy-dispersive detector (HHS5700-VTX-ME7, Hitachi) with electronics capable of handling higher count rates (Xspress3X, Quantum Detectors). Because of the smaller working distance of this optic and the 15° scan direction, this setup is better suited to use with samples with no upstream clearance problems. Sections from biological tissues and organs usually satisfy these requirements. With the shorter working distance of the capillary optic, the sample and detector were in air with no helium chamber. We did not conduct any mechanical vibration evaluations; however, at few-micrometre spatial resolution, no vibration effects were observed in images.

With these two SFXM stations, the beamline 8-BM now has two scanning options. One is for larger samples at modest resolution, but with somewhat higher flux, using the KB optic with larger working distance. The other is for smaller samples using the capillary optic with reduced working distance and flux, but higher spatial resolution. In Section 3, we will see that the fluence per time (photons per area per time) is similar between the two scanning stations. The capillary station is presently in prototype status, with further improvements planned. Biomedical and other users are able to choose which of these two scanning stations are best suited to their research interests without significant downtime for reconfiguring the SFXM system. This is quite useful at a facility where nanofocusing beamlines (which are often oversubscribed) prioritize users needing to collect data on biological samples at sub­micrometre resolution.

The achieved spatial resolution in SFXM depends not only on the properties of the optic but also on the signal and background level for detecting particular chemical elements. While this has been illustrated previously for SFXM imaging (Deng *et al.*, 2015 ▸) and image deconvolution (Deng *et al.*, 2017 ▸), in Section 2 we explore in greater detail the nature of spatial resolution determination using power spectral densities from X-ray fluorescence images. It is shown there that the spatial resolution at a given signal-to-noise ratio (SNR) shows some dependence on how one processes the signal and background.

### 1.3. Absolute photon flux in the two scanning stations

One aspect of characterizing the two SFXM stations was to measure the absolute photon flux Φ(*E*). The APS ran in top-up mode with a constant 100 mA of electron beam current in the storage ring, and we adjusted the beamline optics to maximize the flux observed via an ion chamber just downstream of the beam-defining pinhole at 48.15 m from the source. We carried out absolute photon flux measurements at 10 keV photon energy using a calibrated silicon PIN photodiode (S3590-06, Hamamatsu) immediately downstream of each optic’s focus. These signals were fed into a low-noise current preamplifier (SR570, Stanford Instruments); the measurement scale was confirmed with a voltmeter while the signal was passed to a voltage-to-frequency converter (N101VTF, NOVA) for time-dependent measurements using 8-BM-B’s data acquisition system. To get the most reliable readings, the gain sensitivity *g*_s_ of the preamplifier was tuned to 2 µA V^−1^ for the KB mirror and 1 µA V^−1^ for the capillary. For this particular photodiode, 1 pA through the device corresponded to about 2382 photons s^−1^ at *E* = 10 keV, giving a photon-flux-to-photocurrent conversion factor of *K*(*E*) = 2382 photons pA^−1^ s^−1^. Since the dark current voltage was less than 1% of the beam signal levels, we were able to determine the absolute incident photon flux via 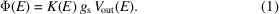
In this way, we found that the KB mirror station, at *E* = 10 keV, delivered a focused flux of Φ = 2.1 × 10^10^ photons s^−1^, while the capillary optic delivered a flux of Φ = 7.7 × 10^9^ photons s^−1^ (in each case, the absolute uncertainty was estimated to be approximately ±5% based on slow variations in the measurements). While for diffraction-limited optics there is a tradeoff between smaller focus and less flux [see, for example, Section 4.4.6 of Jacobsen (2020 ▸)], in this case we expected that the lower flux from the capillary optic was due to its lower aperture with the non-optimized beamline optics as existed for 8-BM (see Section 1.2).

### 1.4. Fluorescence data collection

While there are a variety of sample orientations and detector configurations that can be used in SFXM (Sparks, 1980 ▸; Ryan *et al.*, 2010 ▸; Sun *et al.*, 2015 ▸), most experiments mount the X-ray fluorescence detector at 90° to the incident beam direction so as to minimize elastic and inelastic scattering from horizontally polarized beams at synchrotron light sources (Dzubay *et al.*, 1974 ▸). In addition, the sample and scanning stage motions are typically inclined to the normal of the incident beam by 15° or 45° for a compromise between minimizing X-ray fluorescence self-absorption in planar samples while not introducing too much effective beam broadening in the scanning direction, as illustrated in Fig. 3 ▸. The effective beam width 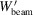 along the scanning stage direction, relative to beam width *W*_beam_ on a sample oriented at normal incidence to the beam, is given by 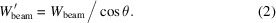
As noted above, we used θ = 15° and θ = 45° for the capillary and KB optics, respectively.

All data shown here were taken with continuous scanning, or ‘fly scan’ mode, where the specimen was moved at near-constant velocity along the fast axis of the scan raster, with data recording advanced over position increments. With the KB scanning system, we frequently examined a large sample area using Δ_*x*_ = Δ_*y*_ = 25 µm, and then acquired higher resolution scans using Δ_*x*_ = Δ_*y*_ = 5–25 µm. With the capillary optic, Δ_*x*_ = Δ_*y*_ = 1–20 µm was typically used. In both cases, per-pixel imaging times of *t*_dwell_ = 20–100 ms were chosen. Both scanning stations have multi-element fluorescence detectors with collimators to minimize the sensitivity to scattering and stray fluorescence from materials other than the sample area in the beam focus. The sum of the signal from all active detector elements was used for data processing. With an incident X-ray beam energy of 10 keV, we were able to estimate the spatially resolved concentrations of biologically important elements including phosphorus, sulfur, potassium, calcium, iron, nickel, copper, and zinc.

To determine the distance from the sample to the X-ray fluorescence detector and thus determine the effective solid angle of collection Ω_eff_ for each detector, we collected average fluorescence intensities *I*_avg_(Δ*d*) at three different detector displacements Δ*d* of 0, 3, and 6 mm along the detection axis. Because the solid angle, and therefore the detected intensity, should vary according to an inverse square law, we were able to determine the effective sample-to-detector plane distance *d*_eff_ using 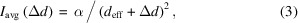
where α is a fitting constant. The strong calcium fluorescence signal from the sample shown in Fig. 1 ▸ was used as the signal, and we obtained effective detector distances of *d*_eff_ = 31.2 mm for the KB system and *d*_eff_ = 14.4 mm in the capillary system. Only *N*_d_ = 3 of the four detector elements in the KB system’s detector were functioning, with each element *n* having active area *A*_act,*n*_ = *A*_act_ = 42.5 mm^2^. With the *N*_d_ = 7 element detector used in the capillary system, the active area for each detector element was 40 mm^2^ as limited by the collimator system. From these values, we determined the effective solid angles of collection Ω_eff_ via 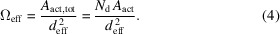
Using this approach, Ω_eff_ = 0.13 sr for the KB system, and Ω_eff_ = 1.35 sr for the capillary system. While these values represent the effective solid angles based on the distances *d*_eff_ determined from equation (3), the actual distances to the detector center involve considering the distances to each detector element their corresponding obliquity factors (see Section S3 of the supporting Information for more details).

For ease of data collection, we developed a graphical user interface (GUI) for the 8-BM EPICS control system used at the APS. This allowed the user to set up a batch of scans for acquisition. The software prompted the user for scan parameters such as scan height and width about a desired center position, pixel size, and the per-pixel exposure time (dwell time). The GUI then calculated the number of pixels per line, and number of scan rows. The software then verified that the parameters for each scan did not exceed motor position or velocity limits, after which it returned an estimated completion time. At the start of each scan in the queue, the software would reset the acquisition hardware and ready the controls hardware such that every *n*th motor pulse produced a single trigger event based on the ratio of the desired pixel size to the motor’s minimum step size. With the XIA xMAP fluorescence detector readout system for the KB mirror scanning station, the full fluorescence spectrum from each element was acquired at each pixel interval. The Xspress3X detector readout electronics used with the capillary optic scanning station provided additional functionality, which required modifications to the data acquisition software. The Xspress3X system was able to internally store full fluorescence spectra for each detector element at each pixel in one scan line, with transfer to the scan control system at the end of each scan line. It also included parameter import/export functionality, event logging, parameter validation, and periodic visual updates indicating that scans were progressing. We calibrated the Xspress3X system as well to ensure accurate and consistent measurement across each of the detector elements and electronics channels prior to commissioning.

### 1.5. Fluorescence data processing

Energy-dispersive X-ray detectors collect all the charge from single above-threshold photon detection events and calculate the photon energy using the electron–hole separation energy of the semiconductor detection surface (for silicon-based detectors, that energy is 3.65 eV) (Shockley, 1961 ▸; Lowe & Sareen, 2007 ▸; Mazziotta, 2008 ▸). However, the collected signal also includes photons that are elastically and inelastically scattered from the sample and also from instrument materials, incomplete charge detection, and escape peaks in the detector material (Van Grieken & Markowicz, 2002 ▸). Therefore, a variety of analysis programs have been developed to process the as-recorded signal, separating X-ray fluorescence photon events from the background (Ryan, 2000 ▸; Vogt, 2003 ▸; Solé *et al.*, 2007 ▸; Schoonjans *et al.*, 2012 ▸; Schoonjans *et al.*, 2013 ▸; Crawford *et al.*, 2019 ▸). These programs return a map of concentration 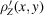 of each element *Z* in units of mass per area. In our case, we used two programs: *MAPS* (Vogt, 2003 ▸) and *M-BLANK* (Crawford *et al.*, 2019 ▸). *MAPS* offers both region-of-interest (ROI) selection (sometimes called ‘spectral binning’), and full-spectrum fitting algorithms. *M-BLANK* offers full-spectrum fitting, while ROI selection is still used in some cases for expediency. Because background subtraction affects both signal and noise levels, one obtains somewhat different resolution estimates for ROI versus full-spectrum fitting when using the power spectral density method described in Section 2. This is discussed in Section S4 of the supporting information.

When obtaining elemental concentration maps 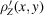 using background subtraction, pixels that are absent of any concentration of a particular element will have Poisson fluctuations in the number of detected fluorescent photons. If the background is measured using strong photon statistics, subtraction of this high-statistics background from low-statistics per-pixel fluorescence measurements when there is little to no elemental content is likely to produce concentration maps 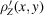 with values fluctuating on either side of zero. This is in fact the case when using analysis programs like *M-BLANK* (Crawford *et al.*, 2019 ▸).

#### 1.5.1. Limit of detection

**Figure 4:**
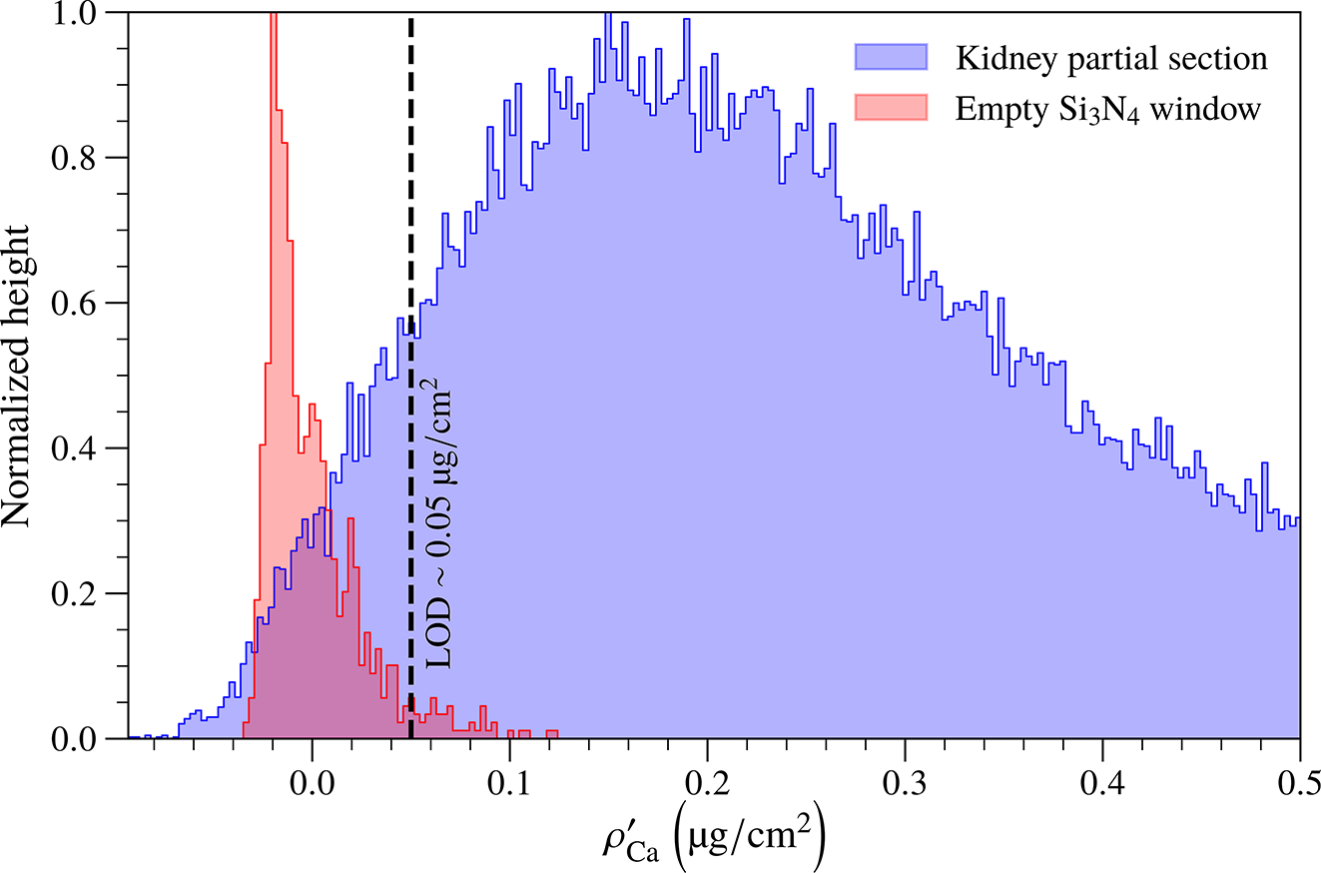
Limit of detection estimation at 8-BM using the capillary optic. We inspected histograms of low-mass concentration pixels 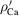 from the Ca image of the kidney partial section of Fig. 1 ▸(*c*) taken at per-pixel dwell time *t*_dwell_ = 50 ms (thus, at an incident fluence of 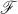 = 2.8 × 10^6^ photons µm^−2^ – see Section 3) and of a photon statistics-corrected scan of an empty Si_3_N_4_ under the assumption of no Ca presence (we increased the distribution width of the histogram of the Si_3_N_4_ window scan by a factor of two for the statistics correction because we scanned it at *t*_dwell_ = 200 ms). We empirically estimated a limit of detection 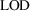 ≃ 0.05 µg cm^−2^ for the kidney partial section since we did not observe that many ‘false-positive’ pixels in the empty Si_3_N_4_ window scan. Our limit of detection was an estimate; a more rigorous statistical approach [see Currie (1968 ▸), for instance] would be needed for a more accurate determination of such a parameter. See Section 2.1 for more details about choosing to look at Ca for the kidney partial section.

Careful determination of limits of detection requires simulation studies of the behavior of full-spectrum fitting methods (like the one employed by *M-BLANK*) with known low- and zero-concentration pixels and the incorporation of Poisson statistics, as well as choices for false-positive and false-negative tolerances (Currie, 1968 ▸). All of that is well beyond the scope of the present study. Instead, we took an empirical approach where we examined a histogram of low-calculated-Ca mass concentration pixels 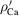 from the kidney partial section shown in Fig. 1 ▸(*c*) (see Section 2.1 for why we chose to look at the Ca map), as well as a histogram of an experimental scan we performed of an empty Si_3_N_4_ window presumably free of calcium (at least as far as this comparison is concerned). Because the Si_3_N_4_ scan utilized a dwell time of *t*_dwell_ = 200 ms as opposed to *t*_dwell_ = 50 ms as used for the kidney specimen scan, we expanded the width of the Si_3_N_4_ window scan by a factor of (200 ms/50 ms)^1/2^ = 2 to correct for differing photon statistics. The resulting histograms shown in Fig. 4 ▸ indicated that at a concentration of 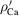 = 0.05 µg cm^-2^, we had very few ‘false-positive’ pixels in the empty Si_3_N_4_ scan; we therefore used this as an estimate of the minimum detectable mass concentration of Ca in our example measurement for *t*_dwell_ = 50 ms (thus, a fluence of 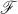 = 2.8 × 10^6^ photons µm^−2^; see Section 3 for more information).

## 2. Resolution determination via power spectral density

One commonly used method for evaluating the spatial resolution of images is to use 3D Fourier shell correlations (or 2D Fourier ring correlations) between two independent images of the same specimen as a function of spatial frequency *u* = 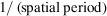 (Saxton & Baumeister, 1982 ▸; Heel & Schatz, 2005 ▸). However, expediency in data collection, as well as radiation dose minimization, make it useful to evaluate spatial resolution from single images. With low-exposure images, this can be carried out using power spectral density (PSD) analysis. In two dimensions, we calculated the PSD *S*(*u*_*x*_, *u*_*y*_) for each element using 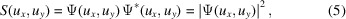
where the spatial frequency representation Ψ(*u*_*x*_, *u*_*y*_) is found in transmission imaging from the complex specimen transmittance ψ(*x*, *y*) via a Fourier transform, or 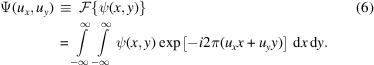
In the discrete Fourier transform with an even number of pixels *N*_*x*_, the spatial frequency *u*_*x*_ runs from −1/(2Δ_*x*_) to +*b*_*x*_/(2Δ_*x*_), with *b*_*x*_ = (*N*_*x*_/2 − 1)/(*N*_*x*_/2) (a similar result holds for *u*_*y*_). When processing images with an odd number of pixels, each image was padded with a row and/or column of the average low signal level. With the exception of the approach described in Section 2.3, we show azimuthally averaged power spectral densities *S*(*u*_*r*_), where 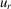 = 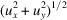 is the radial spatial frequency. We also binned *u*_*r*_ to smaller numbers of pixels to obtain smoother representations.

For objects with no phase contrast, the complex transmittance ψ(*x*, *y*) can be found from the square root of image intensity, or ψ(*x*, *y*) = [*I*(*x*, *y*)]^1/2^. Fluorescence is an incoherent process depending only on sample absorbance, and furthermore the fluorescence spectrum is analyzed to yield a concentration map per element *Z* of 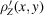 as described in Section 1.5. Normally, we have 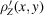 ∝ *I*(*x*, *y*), so we should use 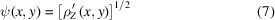
in equations (5) and (6) for power spectral density analysis. However, as noted in Section 1.5, pixels that are absent of any concentration of a particular element will have Poisson fluctuations in the number of detected fluorescent photons, which should lead to fluctuations of 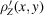 about a value of zero due to subtraction of a background measured with strong photon statistics. The presence of some pixels in 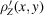 having weak negative values in fact arises when using analysis programs like *M-BLANK* (Crawford *et al.*, 2019 ▸). Therefore, for PSD analysis using equations (5) and (6), we used ψ(*x*, *y*) = 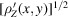 for pixels with positive values of 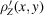 and ψ(*x*, *y*) = 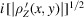 for pixels with negative values of 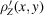.

When shown on a logarithmic scale for power spectral density versus spatial frequency, *S*(*u*_*r*_) typically declines linearly with *u*_*r*_ for many types of images (Porod, 1982 ▸; Jacobsen, 2020 ▸). This corresponds to a signal trend of 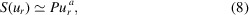
where *a* < 0. When images are obtained using a finite number of photons with associated Poisson noise statistics, it is common for *S*(*u*_*r*_) to decrease until it reaches a constant ‘noise floor’ value *S*_nf_. This is due to Poisson fluctuations being uncorrelated between pixels. As a result, Poisson noise is like a delta function in real space with a ‘flat’ power spectral density that is uniform over all pixels in Fourier space. One can exploit these trends in signal and noise for Wiener noise suppression in Fourier plane representations of images, where the Wiener filter *W*(*u*_*r*_) is given by (Press *et al.*, 1986 ▸) 
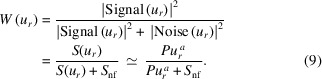
Because *W*(*u*_*r*_) ≃ 1 at low spatial frequencies where *S*(*u*_*r*_) dominates, and because *W*(*u*_*r*_) ≃ 0 at high spatial frequencies where the noise floor *S*_nf_ dominates, it is only in the approximate ‘knee’ region ∼*u*_knee_ [where *S*(*u*_*r*_) ≳ *S*_nf_] that one requires an estimate of the trend of *S*(*u*_*r*_) for Wiener filtering. In this spirit, one can use a fit of 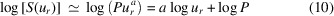
to find the power law decrease slope *a* for the signal. That is, one can use data points from spatial frequencies *u*_*r*_ somewhat lower than the approximate ‘knee’ ∼*u*_knee_ to find the signal trend and thus determine *P* and *a*. One can also use the average of data points at spatial frequencies *u*_*r*_ above the approximate ‘knee’ ∼*u*_knee_ to find 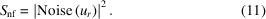
With *P*, *a*, and *S*_nf_ now determined, the actual spatial frequency *u*_knee_ of the ‘knee’ is the value of *u*_*r*_ for which 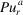 = *S*_nf_, or 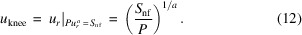
For evaluating the effective spatial resolution of an image, it is best not to have the signal be equal to the noise (the condition of the ‘knee’) but to have the signal be higher than the noise. It is common to use the Rose criterion (Rose, 1946 ▸) of requiring a signal-to-noise ratio of SNR_res_ = 5 for high-quality images (lower SNR values are often deemed acceptable in specific contexts). Thus, we can obtain an estimate of the maximum spatial frequency *u*_res_ corresponding to a high-quality image from the case where the resolution-defining power spectral density is a multiple SNR_res_ of the noise floor *S*_nf_, or 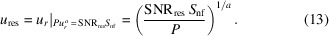
This leads to a spatial resolution estimate of 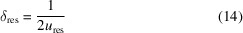
based only upon the power spectral density of a single image.

We developed a Python computer program (using the *PyQt* graphical user interface) called *PyXRFPower* to carry out power spectral density analysis on X-ray fluorescence images. The program allows one to either estimate the spatial resolution assuming a symmetric beam profile or separate resolution in the horizontal and vertical directions (Section 2.3). After reading in a scanning fluorescence X-ray microscope dataset, one can select images corresponding to emission at one or several elemental X-ray fluorescence lines and carry out power spectral density fitting separately for each element. The selection of the data points to use for the signal trend *S*(*u*_*r*_) [equation (8)] and noise floor *S*_nf_ [equation (11)] is accomplished by dragging a cursor through spatial frequency regions on either side of the approximate ‘knee’ ∼*u*_knee_. Spatial frequencies *u*_res_ [equation (13)] resulting from a user-defined value of SNR_res_ and corresponding spatial resolutions are reported alongside data trend fit slopes *a* as provided by equation (8). *PyXRFPower* can currently be found on Github at https://github.com/bwr0835/pyxrfpower.

Using the approaches described above, we characterized the scanning fluorescence X-ray microscopy performance of both the KB mirror and capillary optic experimental stations. For the KB mirror, we acquired images with scan step sizes of Δ_*x*_ = Δ_*y*_ = 5 µm and per-pixel acquisition times of *t*_dwell_ = 25 ms (97.7% live time) and 100 ms (99.6% live time). The scan step size for the capillary optic was Δ_*x*_ = 1 µm and Δ_*y*_ = 2 µm, and *t*_dwell_ = 20 ms (100.0% live time) and 50 ms (99.6% live time). Unless stated otherwise, results shown below were obtained using the *M-BLANK* program (Crawford *et al.*, 2019 ▸) for fluorescence spectrum analysis as described in Section 1.5.

### 2.1. Spatial resolution versus fluorescing element

As noted in Section 1, spatial resolution actually achieved in an image depends on the optic but also on the signal-to-noise characteristics of the X-ray fluorescence image (Deng *et al.*, 2015 ▸; Deng *et al.*, 2017 ▸). We therefore evaluated SFXM images separately for each element using the methods described in Section 2 to find the power spectral density trends for the signal 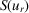 ≃ 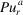 of equation (8) and the noise floor *S*_nf_ of equation (11). From that, spatial resolution estimates δ_res_ of equation (14) for a signal-to-noise ratio of SNR_res_ = 5 were obtained.

**Figure 5:**
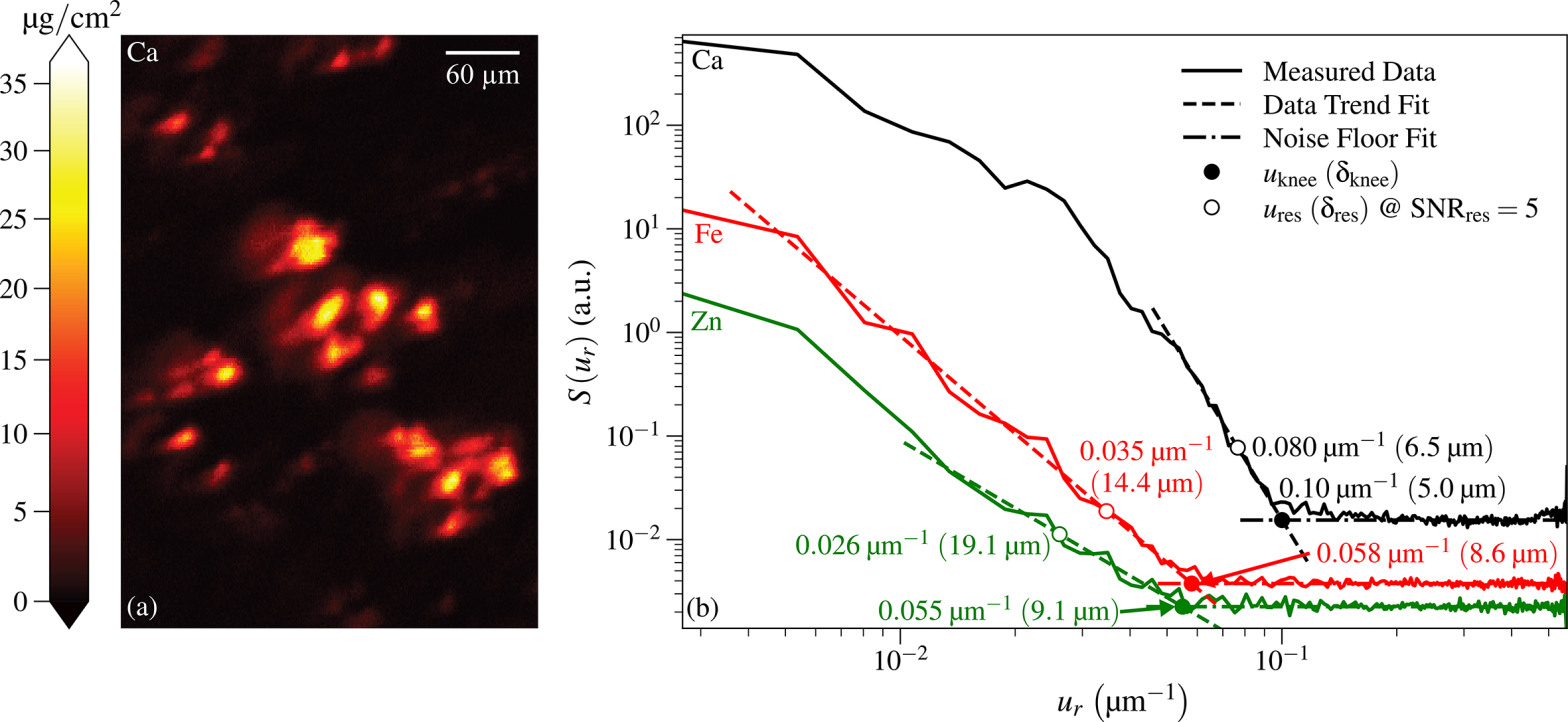
Image of a *Candida albicans*-infected mouse kidney partial section obtained using the capillary optic scan station and a per-pixel scan time of *t*_dwell_ = 50 ms. At left (*a*) is the calcium fluorescence image from Fig. 1 ▸(*c*) as scanned, which is rotated relative to the orientation shown in that figure. At right (*b*) are power spectral density plots for the images of three different elements obtained from the same scan: Ca, Fe, and Zn. Because of differences in concentrations of each of these elements in the sample and differing fluorescence yields and background signals, each element’s image and power spectral density had different signal and noise characteristics. As described in Section 2.1, this leads to different values for the ‘knee’ spatial frequency *u*_knee_ of equation (12) with spatial half-period δ_knee_ = 1/(2*u*_knee_), as well as the spatial frequency *u*_res_ of equation (13) and corresponding spatial resolution δ_res_ of equation (14) for a signal-to-noise ratio of SNR_res_ = 5.

For the capillary optic, we imaged the specimen described in Section 1.1 with 339 × 242 pixels of size Δ_*x*_ = 1 µm and Δ_*y*_ = 2 µm at a per-pixel imaging time of *t*_dwell_ = 50 ms [see Fig. S2(*a*) of Section S2 in the supporting information for the integrated spectrum we collected using this optic]. Of all the elemental images obtained from this sample, the calcium image shown in Fig. 5 ▸(*a*), taken from Fig. 1 ▸(*c*), appeared to have the most favorable signal-to-noise ratio. With a stronger signal, one can come close to the optic-limited resolution that would be observed at low noise conditions. Therefore, we carried out power spectral density analyses on that Ca image, along with images of Fe and Zn fluorescence. The results displayed in Fig. 5 ▸(*b*) show resolution results of δ_res_ = 6.5 µm for Ca, δ_res_ = 14.4 µm for Fe, and δ_res_ = 19.1 µm for Zn [see Fig. S2(*b*) in Section S2 of the supporting information for the Fe and Zn maps alongside the Ca map]. The spatial resolution estimate for the Ca image was in good agreement with a rough estimation obtained using images of a 1951 United States Air Force (USAF) test pattern (R3L3S1P, Thorlabs).

We also scanned a larger area of a region in the same specimen, containing most of the capillary field of view, using the KB mirror scanning station. This scan was over 187 × 75 pixels of size Δ_*x*_ = Δ_*y*_ = 5 µm with a per-pixel imaging time of *t*_dwell_ = 100 ms. This pixel size was just small enough for meeting the Nyquist criterion, and Fig. S4 in Section S4 of the supporting information shows that it was small enough to allow the noise floor to be seen. Using the same analysis approach as shown in Fig. 5 ▸, we obtained resolution estimates of δ_res_ = 10.5 µm for Ca, δ_res_ = 12.8 µm for Fe, and δ_res_ = 12.9 µm for Zn. The weaker Fe and Zn signals came closer to the optic limit in the longer-dwell time KB station measurement than they did in the shorter-dwell time capillary station measurement (which, again, was a different field of view that had smaller concentrations of those two elements), illustrating the point that the achieved spatial resolution depends both on the optic and on signal levels.

When spatial resolution is set by diffraction from an optic’s aperture, the depth of focus is approximated by DOF = 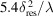 (Jacobsen, 2020 ▸). In that case, focusing to δ_res_ = 6.5 µm by the capillary optic would correspond to DOF = 1.8 mm, while δ_res_ = 10.5 µm would give DOF = 6.8 mm for the KB mirror. However, we did not test for the actual depth of focus in experiments.

### 2.2. Achieved resolution versus scan time

**Figure 6:**
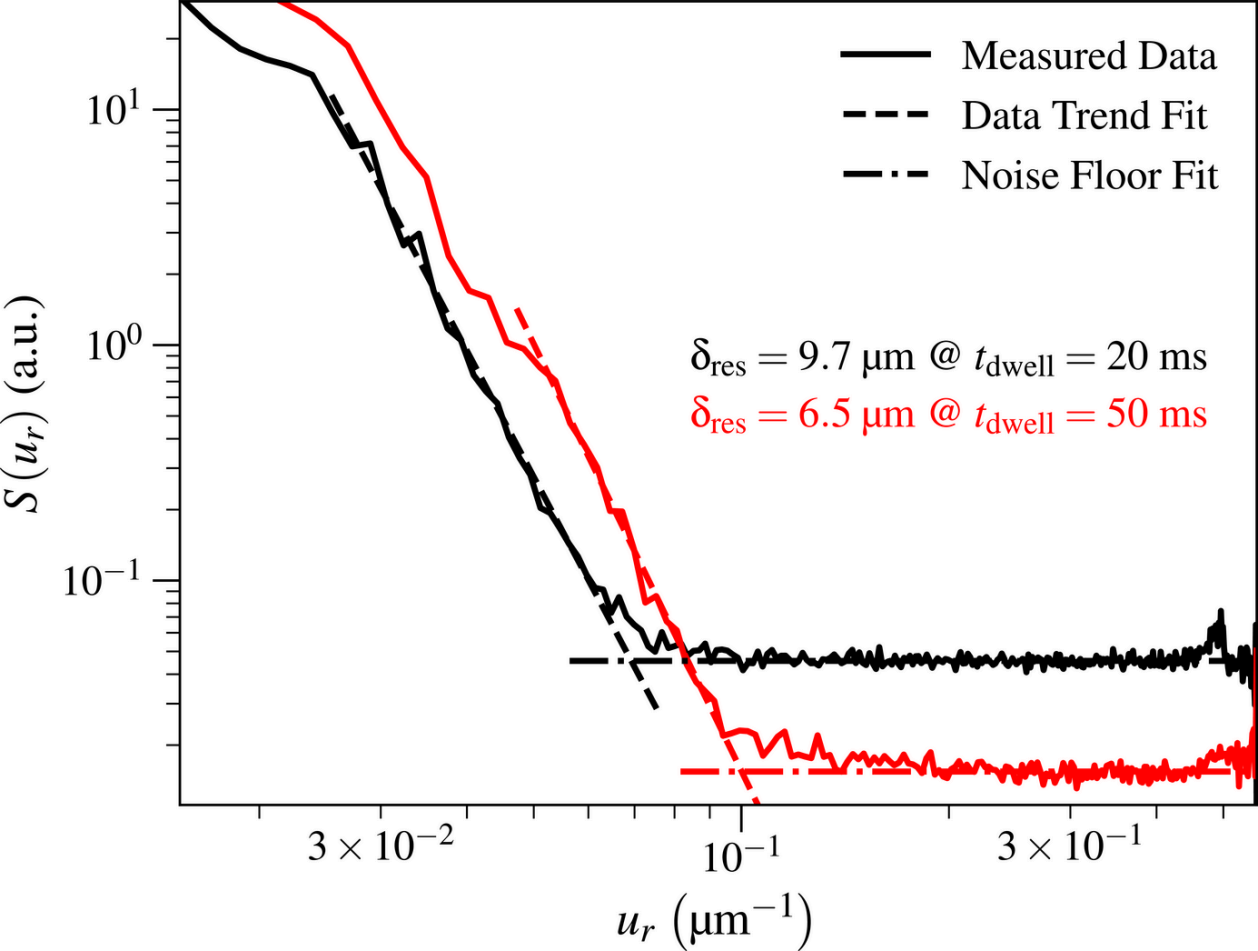
Comparison of isotropic capillary spatial resolutions δ_res_ when increasing per-pixel exposure time *t*_dwell_. The resolution improved from δ_res_ = 9.7 µm at *t*_dwell_ = 20 ms to δ_res_ = 6.5 µm at *t*_dwell_ = 50 ms. The incident photon fluence 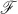 increases in direct proportion with *t*_dwell_ as indicated by equation (16).

As one increases the per-pixel exposure time *t*_dwell_, the signal *S*(*u*_*r*_) of equation (8) should increase relative to the noise floor *S*_nf_ of equation (11), meaning that the obtained spatial resolution δ_res_ of equation (14) should improve as one approaches the high-signal-level limit of the optic. For the Ca image obtained with the KB mirror optic, the resolution at a shortened per-pixel time of *t*_dwell_ = 25 ms was δ_res_ = 15.6 µm, corresponding to about a 52% worsening of spatial resolution compared with the result of δ_res_ = 10.2 µm at *t*_dwell_ = 100 ms. Because the spatial resolution limit involves both the intrinsic resolution of the optic and the signal-to-noise ratio, it does not scale simply with exposure time alone. For the Ca image obtained with the capillary optic, decreasing the per-pixel exposure time to *t*_dwell_ = 20 ms gave δ_res_ = 9.7 µm compared with δ_res_ = 6.5 µm at *t*_dwell_ = 50 ms, or about a 32% worsening of spatial resolution. These results clearly show how the achieved spatial resolution depends strongly on exposure time for photon statistics-limited images such as are obtained in scanning fluorescence X-ray microscopy of intrinsic concentrations of metals in biological tissues. Fig. 6 ▸ shows the two *S*(*u*_*r*_) profiles we obtained for the capillary at the two values of *t*_dwell_ corresponding to that optic.

### 2.3. Non-azimuthally symmetric spatial resolution estimate

**Figure 7:**
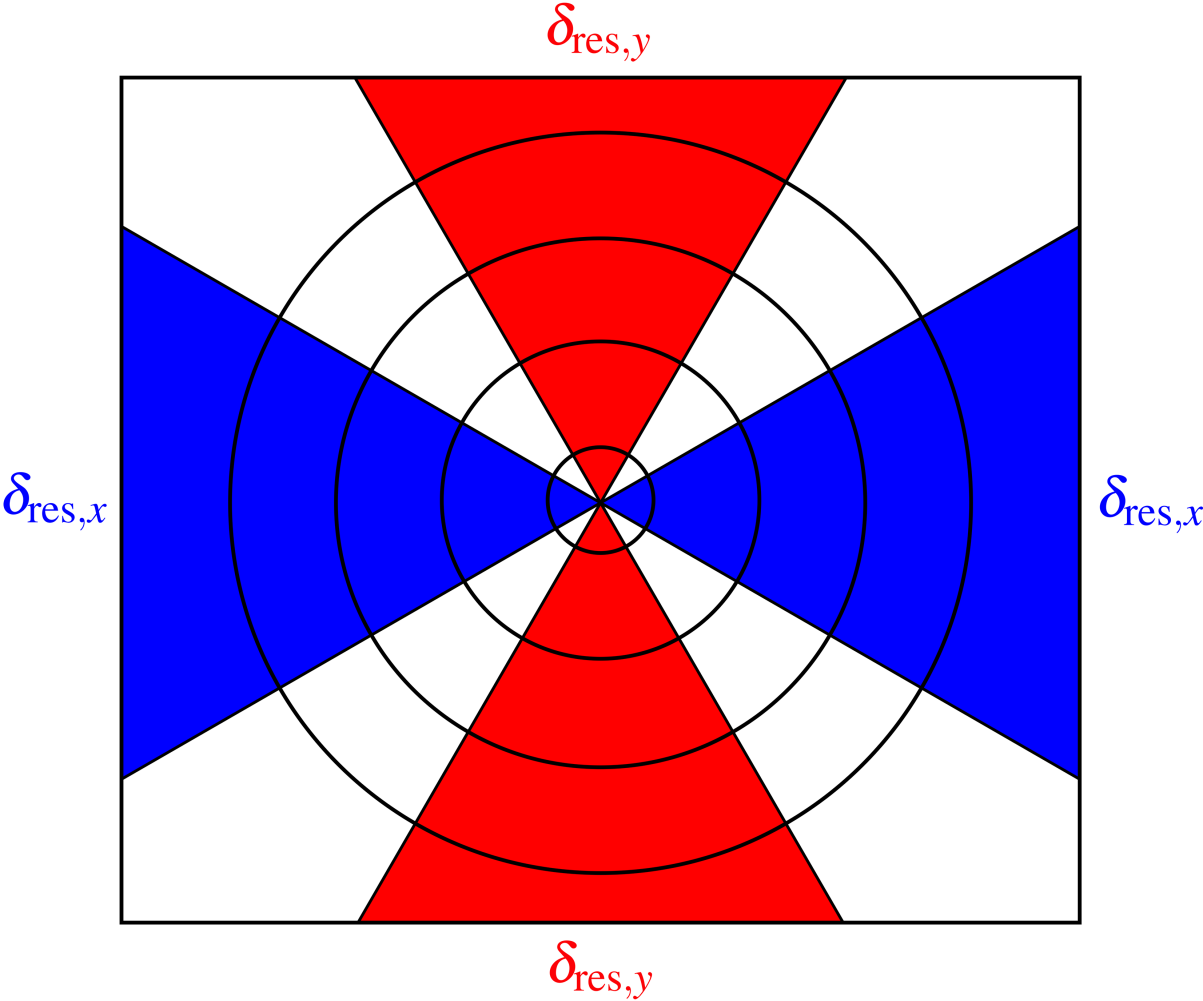
When images have asymmetry in their spatial resolution, one can define azimuthal angle ranges about the *x* and *y* axes (shown here as blue and red regions, respectively) to obtain separate estimates of spatial resolution of δ_res,*x*_ and δ_res,*y*_. We used azimuthal angle ranges of ±30° about the *x* and *y* axes as a compromise between the mixing of *u*_*x*_ and *u*_*y*_ components and having too few ‘spatial frequency pixels’ in each Fourier direction for each circle being averaged over.

Up to this point, we have assumed that the spatial resolution is the same in the horizontal and vertical directions. However, it is not uncommon at synchrotron light source beamlines to have slight spatial resolution asymmetries due to both beamline optics, and tilt of the sample and scanning stage motion as shown in Fig. 3 ▸. One can therefore modify the above analysis so that it is carried out in two distinct azimuthal angle ranges as shown in Fig. 7 ▸, with azimuthal angle ranges of ±30° about each direction representing one reasonable choice. This allows for resolution estimates of δ_res,*x*_ and δ_res,*y*_, respectively.

**Figure 8:**
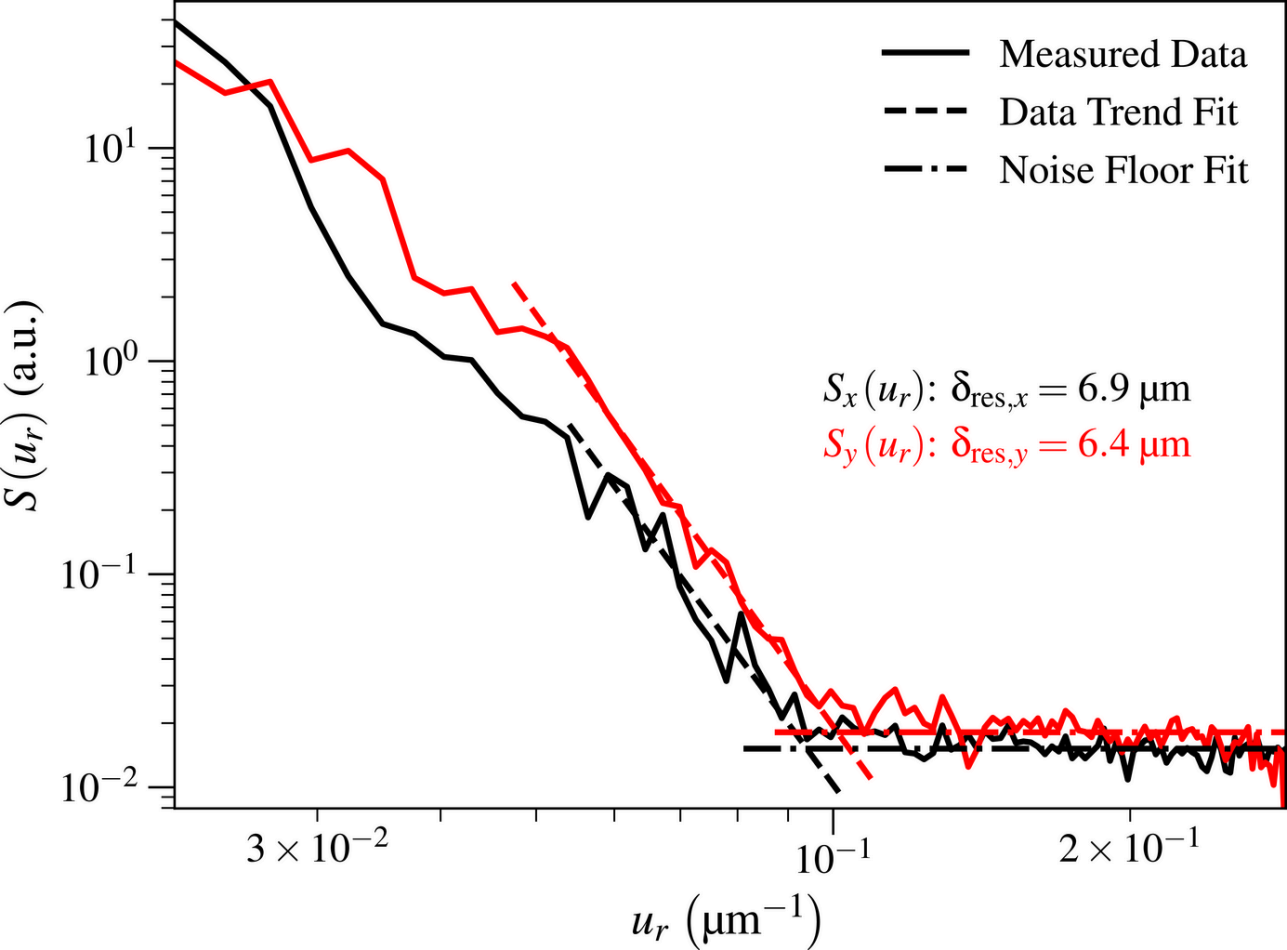
Using the same Ca image as shown in Fig. 5 ▸(*a*), we separately calculated power spectral densities *S*_*x*_(*u*_*r*_) and *S*_*y*_(*u*_*r*_) with the method shown in Fig. 8 ▸ with angular ranges of ±30° about the *x* and *y* axes, respectively. This yielded spatial resolution estimates of δ_res,*x*_ = 6.9 µm and δ_res,*y*_ = 6.4 µm, whereas the azimuthally averaged spatial resolution result in Fig. 5 ▸ was δ_res_ = 6.5 µm. We truncated the maximum value of *u*_*r*_ shown here to 0.29 µm^−1^. The spatial resolution asymmetry might have been due to a slight tilt misalignment of the capillary optic, but we did not systematically explore that.

For the Ca image shown in Fig. 5 ▸(*a*) for which the azimuthally averaged resolution was δ_res_ = 6.5 µm, we used the approach shown in Fig. 7 ▸ to obtain separate estimates for the spatial resolution in the *x* and *y* directions, yielding δ_res,*x*_ = 6.9 µm and δ_res,*y*_ = 6.4 µm as shown in Fig. 8 ▸. Because the maximum value of *u*_*y*_ decreased by a factor of two due to using Δ_*y*_ = 2 µm step size (versus Δ_*x*_ = 1 µm), *S*_*x*_(*u*_*r*_) did not extend out to the same value of *u*_*r*_ as *S*_*y*_(*u*_*r*_). However, in both cases the step size was smaller than half the spatial resolution, thus satisfying the Nyquist sampling criterion. For the KB mirror, where the azimuthally averaged Ca image resolution was δ_res_ = 10.5 µm, the resolution in the two directions was δ_res,*x*_ = 9.5 µm and δ_res,*y*_ = 12.1 µm.

### 2.4. Sensitivity in resolution estimation due to selection of data trend points

**Figure 9:**
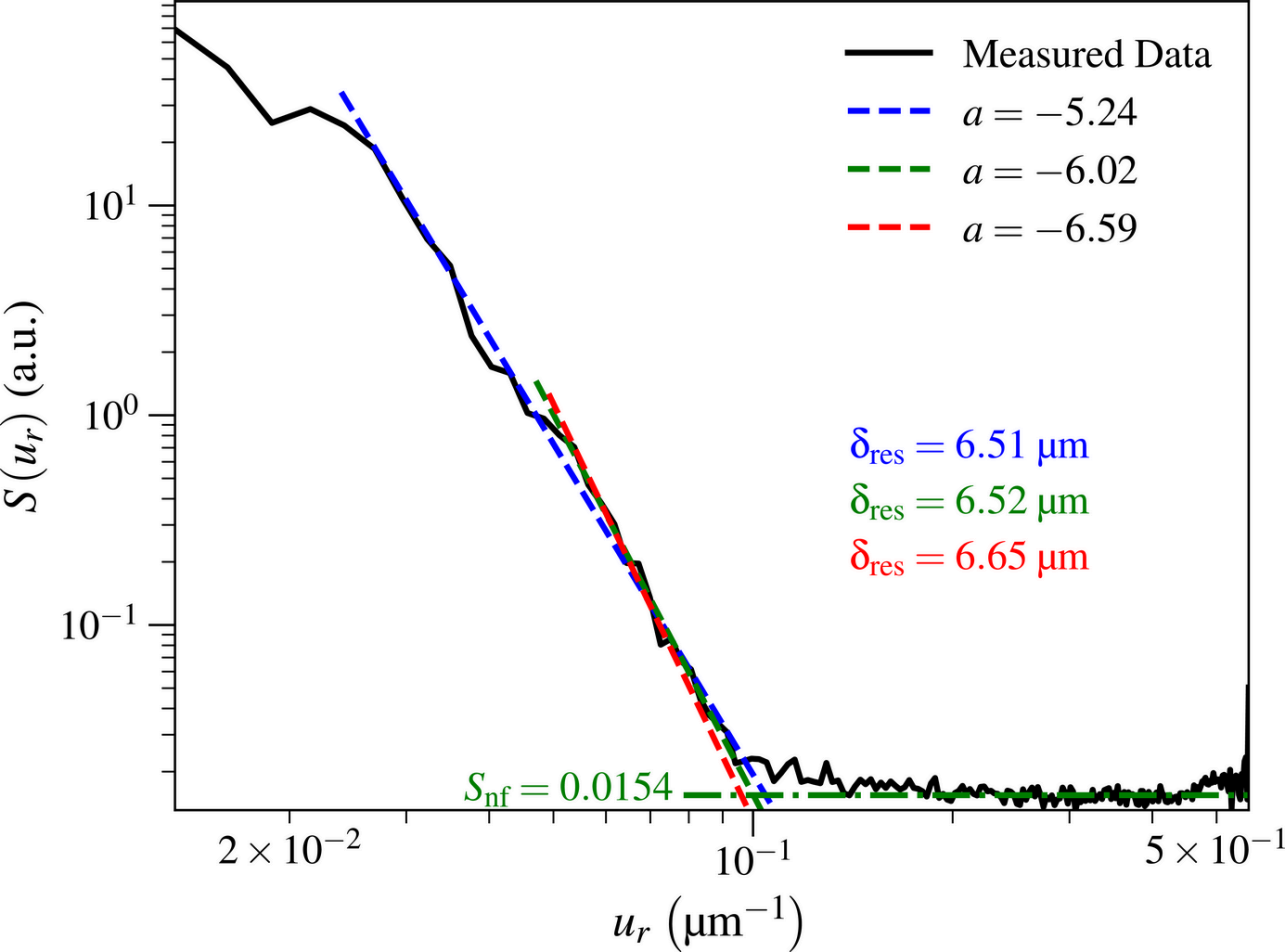
Test of the dependence on the estimated spatial resolution δ_res_ based on three different selections of the ‘signal’ trend points to include in the fit of 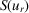

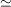

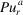
 of equation (8), as discussed in Section 2.4. We carried this test out on the Ca image shown in Fig. 5 ▸(*a*). These three different estimates for the spatial resolution δ_res_ of equation (14) were within 2.2% of each other.

Our method of evaluating spatial resolution using power spectral density in photon-limited images relies on fits of the ‘signal’ region of the power spectral density using 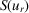 ≃ 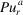 from equation (8), and the ‘noise’ floor *S*_nf_ from equation (11). As noted above, the ‘signal’ points should be selected over some range of spatial frequencies below the estimated ‘knee’ ∼*u*_knee_, while the ‘noise’ floor should be obtained by averaging points above ∼*u*_knee_. In order to test the sensitivity of the spatial frequency *u*_res_ of equation (13) and the resulting resolution estimate δ_res_ of equation (14) to user selections, we show the results for three different selections of ‘signal’ spatial frequencies and resulting values of slope *a* and resolution δ_res_ in Fig. 9 ▸. For the Ca image of Fig. 5 ▸(*a*), we obtained slopes of *a* = −5.23, *a* = −6.02, and *a* = −6.59 with respective resolution estimates of δ_res_ = 6.51 µm, δ_res_ = 6.52 µm, and δ_res_ = 6.65 µm. Even though the slope estimates *a* were quite different from each other, the spatial resolution estimates were consistent within a range of 2.2%. For the KB mirror image with *t*_dwell_ = 100 ms, a similar analysis yielded δ_res_ = 10.30 µm, δ_res_ = 10.49 µm, and δ_res_ = 10.26 µm, again consistent with a spatial resolution estimate range of 2.2% (the respective slopes were *a* = −4.65, *a* = −5.47, and *a* = −6.32). We therefore infer that our approach allows one to estimate the achieved spatial resolution in these X-ray fluorescence images with a reproducibility of better than 3%.

## 3. Fluence per time on samples

The achievable spatial resolution in low-photon statistics images is limited in part by the fluence 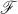 on the sample, or the cumulative photons per area (Schropp & Schroer, 2010 ▸; Deng *et al.*, 2015 ▸; Deng *et al.*, 2017 ▸; Jacobsen, 2020 ▸), as shown in Fig. 6 ▸. We approximated the area of the probe *A*_beam_ as 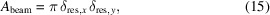
since, for a symmetric Airy probe, the spatial resolution is equal to the radius associated with the first minimum. With that in mind, we can use the absolute photon fluxes measured in Section 1.3 and the best resolution as obtained from Ca images (Section 2.3) to estimate the fluence per time 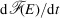 as 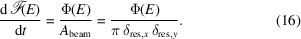
Utilizing this approach, fluence per time estimates of 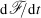 = 5.8 × 10^7^ photons µm^−2^ s^−1^ ± 6.6% for the KB mirror optic and 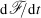 = 5.6 × 10^7^ photons µm^−2^ s^−1^ ± 6.6% for the capillary were obtained.

## 4. Conclusion

We have modified the 8-BM-B beamline at the Advanced Photon Source (APS) at Argonne National Laboratory to provide two different setups for scanning fluorescence X-ray microscopy (SFXM) studies of intrinsic metals in biological tissue sections. The KB mirror station provides a spatial resolution of 10.5 µm (best used for larger area scans), while the prototype capillary optic station provides an improved spatial resolution of 6.5 µm as shown in Section 2.1. We have described the use of power spectral density analysis of single images to obtain spatial resolution estimates, and we have shown how the achieved spatial resolution is affected by signal strength from different fluorescing elements.

The results reported in this work were for the beamline as it existed right before the shutdown of the original APS storage ring in April 2023. The upgrade of the APS (APS-U) (Kerby, 2023 ▸) should offer a slight improvement in the brightness of bending magnet sources (and 75–100× improvement for undulator sources). Further gains might be possible by addressing limitations in the beamline toroidal mirror discussed in Section 1.2. Therefore, this beamline should be an even more valuable resource for future studies of elemental distribution in biological tissues, aiding user communities including that of the NIH-supported QE-MAP center at Michigan State University.

## 5. Related literature

The following reference, not cited in the main body of the paper, has been cited in the supporting information: Qiao *et al.* (2020 ▸).

## Supplementary Material

Raw spectral data and additional elemental maps for the capillary, capillary beam spot size evaluation at beamline 32-ID at the Advanced Photon Source, detector obliquity and solid angle, and effect of x-ray fluorescence spectrum fitting method on spatial resolution. DOI: 10.1107/S1600577526000925/tol5019sup1.pdf

## Data Availability

Elemental concentration maps as fitted and mass-calibrated by *M-BLANK* are available on Arch at https://doi.org/10.21985/n2-cx5v-f797. Any spectra of interest are available upon request.
