# Supplementary material for: Multifunctional bending magnet beamline with a capillary optic for X-ray fluorescence studies of metals in tissue sections
Source: J Synchrotron Radiat. 2026 Feb 19;33(Pt 2):319–30. doi: 10.1107/S1600577526000925 (PMC12948027; doi:10.1107/S1600577526000925)
Supplement: Supplementary file 1 [file s-33-00319-sup1.pdf]

JOURNAL OF SYNCHROTRON RADIATION

Volume 33 (2026)

Supporting information for article:

Multifunctional bending magnet beamline with a capillary optic for X-ray fluorescence studies of metals in tissue sections

Benjamin, Andrew M., Qiaoling, Arthur T., Barry, Fabricio S., Evan, Valeria C., Asia S., Naisargi K., Thomas V., Chris, Benjamin, Andrew, Qiaoling, Arthur, Barry, Fabricio, Evan, Valeria, Asia, Naisarghi, Thomas and Chris

Supporting information for the article:

Multifunctional bending magnet beamline with a capillary optic for X-ray fluorescence studies of metals in tissue sections

Benjamin Roter[a,*], Andrew M. Crawford[b,c,*], Qiaoling Jin[d,e], Arthur T. Glowacki[f], Barry Lai[f], Fabricio S. Marin[f], Evan Maxey[f], Xianbo Shi[f], Valeria C. Culotta[g], Asia S. Wildeman[g], Naisargi K. Patel[g], Thomas V. O'Halloran[b,c,h,†], and Chris Jacobsen[d,a,e,†]

[*]These authors contributed equally to this work.

[†]Corresponding authors

[a]Applied Physics Program, Northwestern University, Evanston, IL 60208, USA, [b]Department of Microbiology, Genetics & Immunology, Michigan State University, East Lansing, MI 48824, USA, [c]Department of Chemistry, Michigan State University, East Lansing, MI 48824, USA, [d]Department of Physics and Astronomy, Northwestern University, Evanston, IL 60208, USA, [e]Chemistry of Life Processes Institute, Northwestern University, Evanston, IL 60208, USA, [f]X-ray Science Division, Advanced Photon Source, Argonne National Laboratory, Lemont, IL 60439, USA, [g]Department of Biochemistry and Molecular Biology, Bloomberg School of Public Health, Johns Hopkins University, Baltimore, MD 21205, USA, [h]Elemental Health Institute, Michigan State University, East Lansing, MI 48824, USA

S1. Characterization of capillary focusing capability

Section 1.2 of the main document noted that the capillary optic's resolution with optimum illumination was much better than what was observed using the illumination available at APS (Advanced Photon Source) beamline 8-BM-B as it existed before the APS-Upgrade [the upgrade is discussed in (Kerby, 2023)]. We outline here the optical tests of the capillary carried out using the small source size and divergence of undulator beamline 28-ID-B at the APS. The schematic of the experimental setup is shown in Fig. S1(*a*), and the capillary parameters are listed in Table S1.

Table S1: Designed and factory-achieved capillary parameters.

	Specification	Achieved
Source-to-focus distance (mm)	2027	2027
Semi-minor axis (mm)	1.075	1.072
Capillary length (mm)	50	50.3
Entrance aperture diameter (mm)	0.785	0.786
Exit aperture diameter (mm)	0.425	0.424
Working distance (mm)	20.0	20.1

APS beamline 28-ID-B uses an undulator with a period of 3.3 cm and a total length of 2.3 m. We employed a double-multilayer monochromator (DMM) and tuned it to an energy of 13 keV with a spectral bandwidth of 0.01%. The white beam slit, with dimensions of (0.1 mm)×(0.1 mm), functioned as the source. We used a polymer compound refractive lens (CRL) to image the white beam slit to a focal plane at 0.1 m downstream. According to prior quality measurements of the polymer CRL (Qiao *et al.*, 2020), the focal spot (acting as the

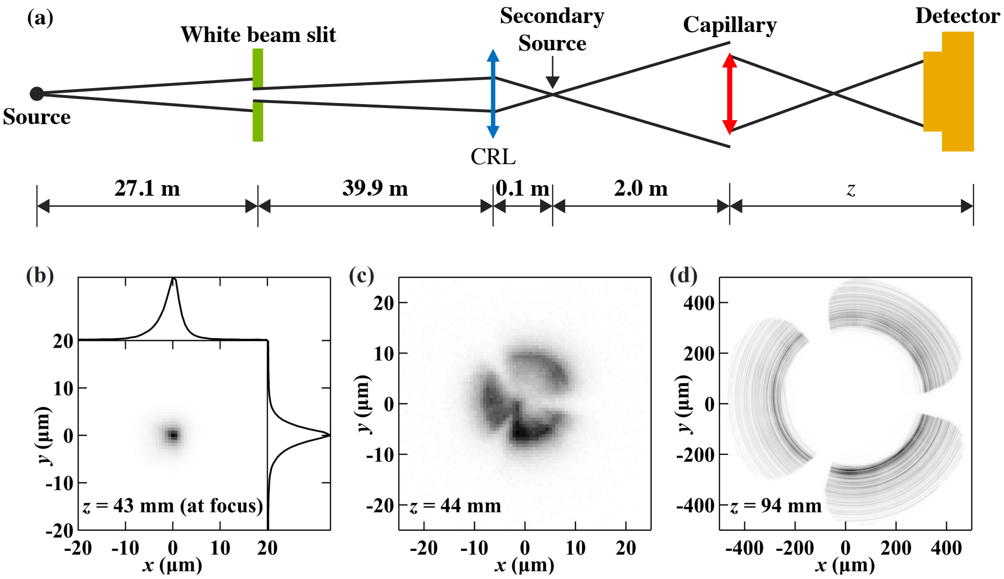

Figure S1: Schematic of the 28-ID-B experimental setup (*a*) for measuring the focus capability of the capillary. Beam profiles were recorded at (*b*) z = 43 mm, (*c*) z = 44 mm, and (*d*) z = 94 mm from the downstream end of the capillary.

secondary source) was estimated to be smaller than 1 µm when considering the geometric demagnification and the CRL aberrations. We positioned the test capillary 2.0 m downstream from the secondary source, with the expected focal plane located about 45 mm beyond the capillary center. This configuration aimed to produce a geometrical image size under 25 nm, predicated on a secondary source dimension of 1 µm and the assumption of ideal focusing optics. Consequently, any focal size measurements surpassing this threshold would likely result from aberrations within the capillary. Beam profiles were profiles using a detector system composed of a 100 µm thick LuAG:Ce scintillator, a 10× objective lens, and a Zyla sCMOS camera (Andor); this scintillator camera system achieves an effective pixel size of 0.65 µm and a spatial resolution of 2.2 µm when accounting for the detector point spread function (PSF).

We identified the optimal focal spot to be at z = 43 mm downstream from the capillary center, with a FWHM size of (3.6 µm)×(3.7 µm), as shown in Fig. S1(*b*). Assuming that this beam size was combined in an RMS sense with the spatial resolution of the scintillator–camera system, the actual focal spot size of the capillary optic, we estimate, is (2.9 µm)×(3.0 µm) FWHM, assuming a Gaussian beam distribution. This estimated size effectively represents the point spread function of the capillary. The primary factor contributing to the focal spot size was the capillary's internal surface slope error, with an average RMS slope error of 14 µrad. These results were consistent with predictions made using the surface figure data provided by the capillary manufacturer. In Figs. S1(*c*) and S1(*d*), we show the beam profiles captured at positions z = 44 mm and z = 94 mm downstream from the capillary center, respectively. The observed three-fold symmetry arises from the capillary's support structure, which partially obstructs the beam. The circular striations visible in Fig. S1(*d*) provide clear evidence of figure errors on the internal surface of the capillary.

S2. Integrated spectra and additional capillary x-ray fluorescence maps

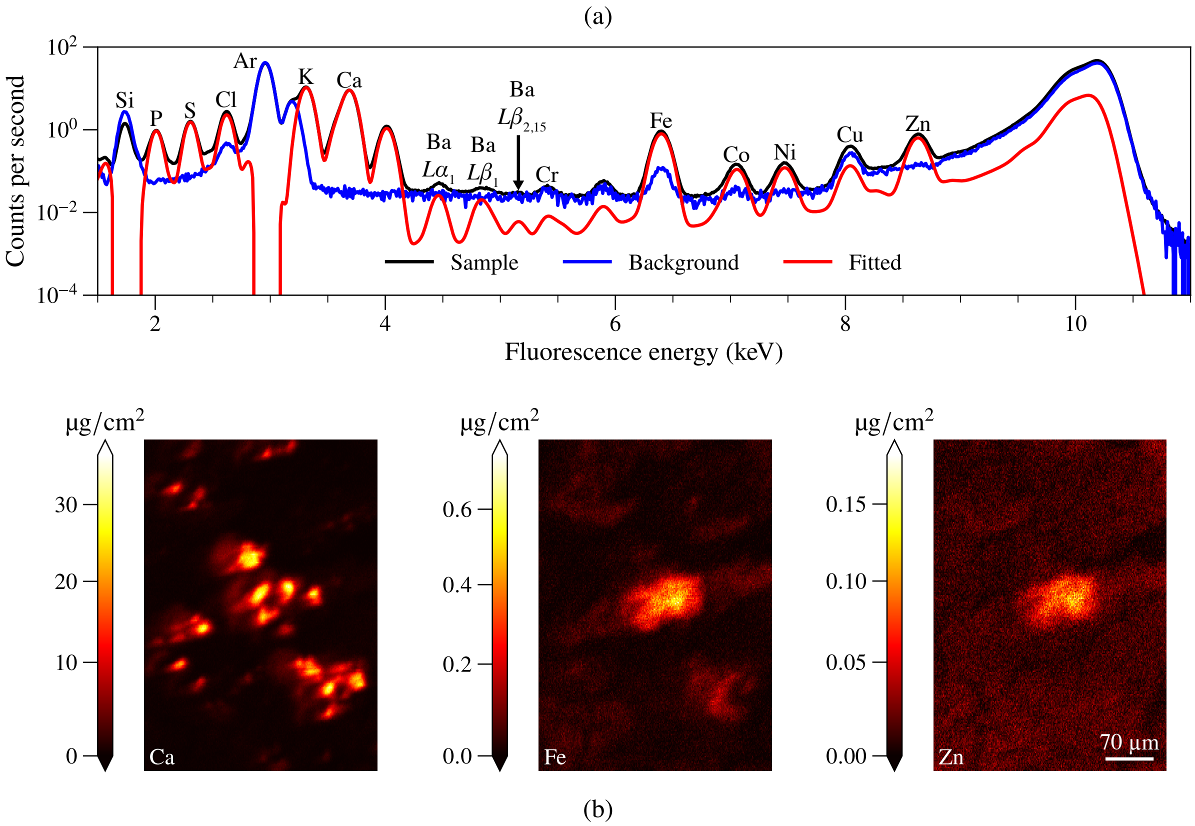

Figure S2: X-ray fluorescence spectrum (*a*) corresponding to the sum of all pixels in the scanning fluorescence x-ray microscopy images we obtained for different elements. The Ca, Fe, and Zn maps are shown in (*b*) as mass concentrations $\rho'_Z(x, y)$. This scan was for an area of a mouse kidney sample examined using the capillary optic experimental station using a per-pixel imaging time of $t_{\mathrm{dwell}} = 50$ ms. (The spectra and images pertain to the signal-dependent resolution discussion and results of Section 2.1 and Fig. 5 in the main document.) In (*a*), the apparent negative Si signal in the fitted spectrum arose due to attenuation of sample signal in the overlying Si_3N_4 window. The apparent negative signal for Ar represented subtle signal fluctuations (less than 5%) around zero; these fluctuation occurred due to background subtraction of a zero signal in the presence of noise. Power spectral density analysis yielded estimates for the achieved spatial resolutions in the images of (*b*) [see Fig. 5(*b*)] of $\delta_{\mathrm{res}} = 6.3$, 14.4, and 19.1 μm for Ca, Fe, and Zn, respectively.

S3. Solid angle coverage in planar multi-element x-ray fluorescence detectors

In Section 1.4 of the main manuscript, we reported effective solid angle coverages of $\Omega_{\mathrm{eff}} = 1.35$ sr and $\Omega_{\mathrm{eff}} = 0.13$ sr for the 7- and 4- element energy-dispersive x-ray fluorescence detectors used with the capillary and KB mirror scanning stations, respectively. Those values were calculated under the ideal assumption of a detector (total active area $A_{\mathrm{act,tot}}$) on a spherical surface of radius d_{eff} (the distance from the fluorescence emitter to the sample), leading to the expected inverse square law dependence of detected signal versus d_{eff}. The effective specimen-to-detector distances found using these assumptions were $d_{\mathrm{eff}} = 14.4$ mm for the

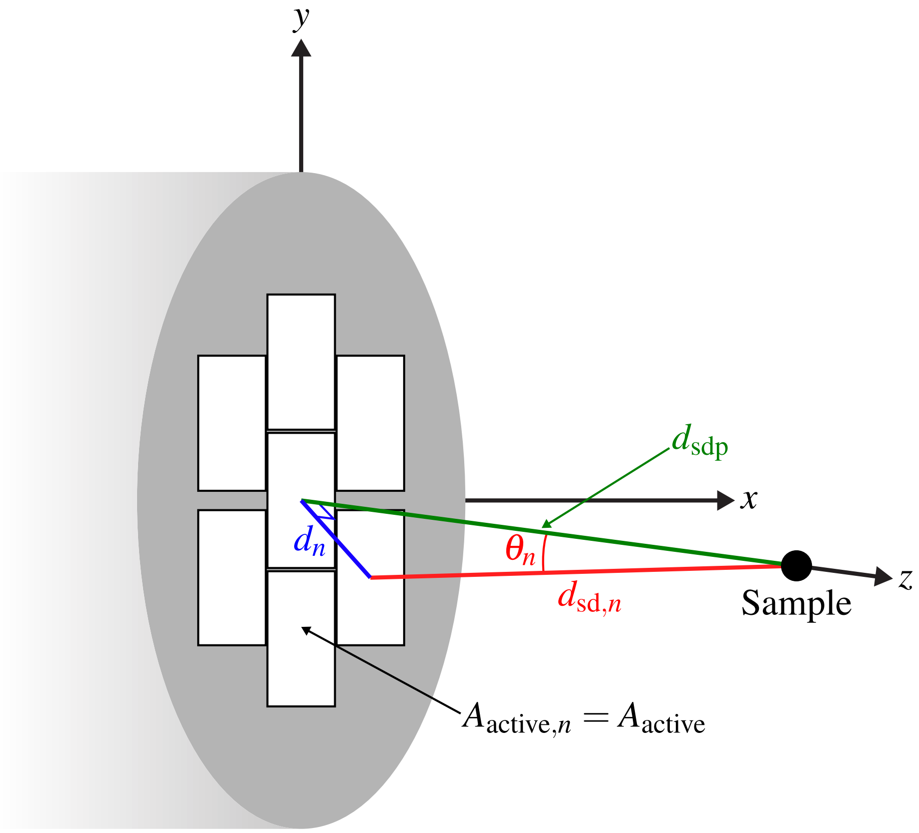

Figure S3: Schematic of a 7-element planar detector facing a fluorescence emitter located at a distance d_{sdp} from the center of the detector plane. An off-axis detector element is at a larger distance $d_{sd,n}$ from the emitter as given by equation (S1). In addition, not oriented perpendicular to the emitter; its effective area is modified by an obliquity factor $\cos\theta_n$ given by equation (S2).

capillary system, and $d_{eff} = 31.2$ mm for the KB mirror system.

In reality, multi-element detectors are usually fabricated with each detector element on the same plane. As a result, some of the N_d total detector elements will be slightly farther away from the fluorescence emitter than an element at the center. Furthermore, since they are on a plane, they are not tilted towards the emitter, meaning that one must account for their oblique orientation. This is shown schematically in Fig. S3. Let d_{sdp} represent the actual distance along the z axis from the emitter to the detector plane center. If the nth detector element of area A_n is radially offset in the plane of the detector by a distance d_n, its net distance $d_{sd,n}$ from the source is

$$d_{sd,n} = \sqrt{d_n^2 + d_{sdp}^2}. \tag{S1}$$

Its solid angle coverage will be affected by its orientation at an oblique angle θ_n, which can be found from

$$\cos\theta_n = \frac{d_{sdp}}{d_{sd,n}}. \tag{S2}$$

The factor $\cos\theta_n$ is known as the obliquity factor. As a result, the effective solid angle Ω_{eff} of the multi-

element detector is given by

$$\Omega_{\text{eff}} = \sum_{n=0}^{N_d-1} \frac{A_{\text{act},n}}{d_{\text{sd},n}^2} \cos\theta_n = \frac{A_{\text{act,tot}}}{d_{\text{eff}}^2}, \tag{S3}$$

where the last expression is the solid angle that would result from all detector elements both being oriented at normal incidence to the emitter and being located at the same distance d_{eff} from the emitter (effectively the approximation of a spherical detector). We can therefore find $1/d_{\text{eff}}^2$ according to

$$\frac{1}{d_{\text{eff}}^2} = \sum_{n=0}^{N_d-1} \frac{A_{\text{act},n}}{A_{\text{act,tot}}} \frac{\cos\theta_n}{d_{\text{sd},n}^2} = \sum_{n=0}^{N_d-1} \frac{A_{\text{act},n}}{A_{\text{act,tot}}} \frac{d_{\text{sdp}}}{\left(d_n^2 + d_{\text{sdp}}^2\right)^{3/2}}. \tag{S4}$$

If each detector element has the same active area $A_{\text{act},n} = A_{\text{act}}$, this simplifies to

$$\frac{1}{d_{\text{eff}}^2} = \frac{1}{N_d} \sum_{n=0}^{N_d-1} \frac{d_{\text{sdp}}}{\left(d_n^2 + d_{\text{sdp}}^2\right)^{3/2}}. \tag{S5}$$

Thus, if we know the radial offset distances d_n for each detector element, we can use our measurement of d_{eff} to solve for the physical distance d_{sdp} from the emitter to the center of the planar detector numerically using equation (S5).

For the 7-element detector at the capillary setup with $d_{\text{eff}} = 14.4$ mm, we found the physical distance to the center of the detector plane to be $d_{\text{sdp}} = 9.8$ mm. For the 4-element detector at the KB mirror setup with $d_{\text{eff}} = 31.2$ mm, we obtained $d_{\text{sdp}} = 29.6$ mm.

S4. Achieved resolution versus spectral analysis method

As noted in Section 1.5 of the main document, the signal recorded by energy-dispersive detectors used in scanning fluorescence x-ray microscopy involves factors such as x-ray scattering from the specimen and also from accidental illumination of instrument hardware (which can in turn lead to x-ray fluorescence emission), and additional effects such as incomplete collection of the charge from a photon absorbed in the detector. The most basic approach to fluorescence signal analysis is simply to define spectral regions of interest around each fluorescence peak and treat the number of fluorescence photons detected as the total photon count within this spectral ROI (for example, a region covering the FWHM of a peak). This approach makes the simplifying assumption that the background is negligible compared to the photon count from strong fluorescence lines, and it provides rapid results for immediate evaluation. However, most scanning fluorescence x-ray microscopy experiments now make use of spectral fitting methods as described in Section 1.5 in the main manuscript, where both signal and background distributions are estimated so that the background can be subtracted from the signal. The choice of spectral binning versus full-spectrum fitting leads to different characteristics for the mass concentration $\rho'_Z(x, y)$, and thus to $\psi(x, y) = \sqrt{\rho'_Z(x, y)}$ [equation (7) in the main text], used for spatial resolution estimation based on power spectral density analysis. This will consequently affect both the power law fit Pu_r^a to the signal of equation (8) and the estimated noise

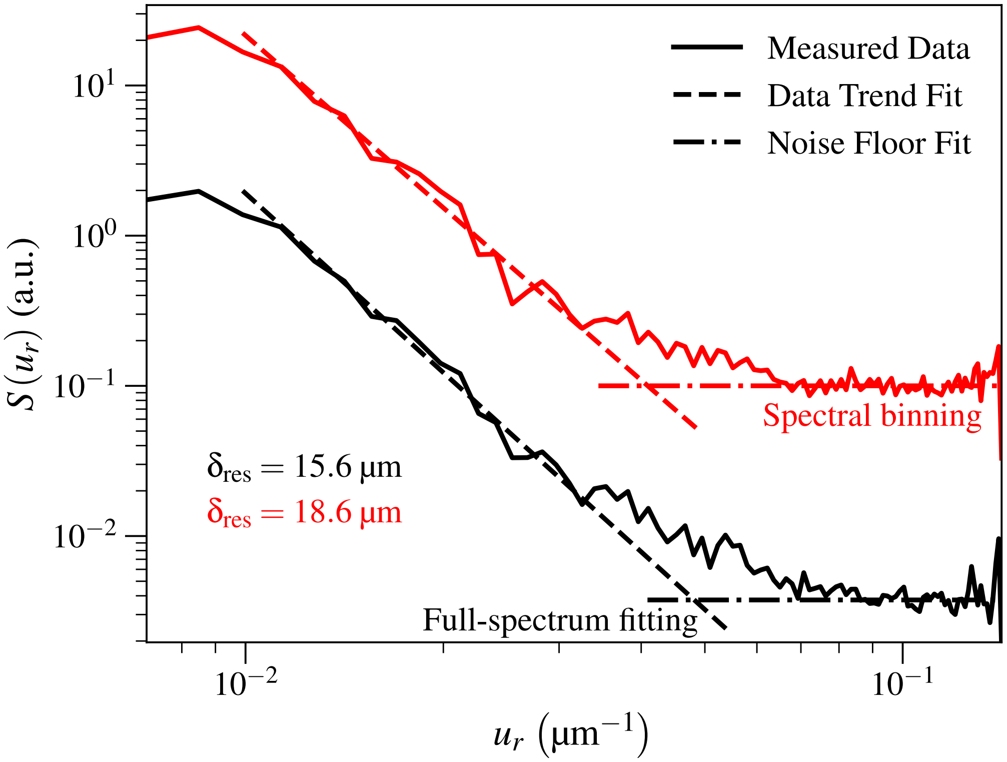

Figure S4: Effect of different approaches to x-ray fluorescence spectrum analysis on the achieved spatial resolution as evaluated using power spectral density. Shown here are power spectral densities and fits for $S(u_r)$ of equation (8) and noise floor S_{nf} of equation (11) for the Ca fluorescence dataset obtained using the KB mirror optic with $t_{\mathrm{dwell}} = 25$ ms. We used two different fluorescence spectrum analysis approaches: simple spectral binning, versus full-spectrum fitting where a large-statistics background signal is subtracted before integrating fitted fluorescence peaks (Crawford *et al.*, 2019). The estimate for achieved spatial resolution δ_{res} of equations (12) and (14) improved from $\delta_{\mathrm{res}} = 18.6$ μm with the spectral binning method to $\delta_{\mathrm{res}} = 15.6$ μm with the full spectrum fitting method.

floor S_{nf} of equation (11), and thus the spatial resolution estimate δ_{res} obtained using equations (13) and (14). In Fig. S4, we show the power spectral density for the same Ca dataset measured with a short per-pixel acquisition time of $t_{\mathrm{dwell}} = 25$ ms using the KB scanning station, but analyzed using two different approaches: spectral binning as is available in MAPS (Vogt, 2003) versus full-spectrum fitting as offered by programs like MAPS and M-BLANK (Crawford *et al.*, 2019), with M-BLANK used here.

References

Crawford, A. M., Deb, A. & Penner-Hahn, J. E. (2019). *Journal of Synchrotron Radiation*, **26**(2), 497–503.
Kerby, J. (2023). *Synchrotron Radiation News*, **36**(4), 26–27.
Qiao, Z., Shi, X., Kenesei, P., Last, A., Assoufid, L. & Islam, Z. (2020). *Review of Scientific Instruments*, **91**, 113703.
Vogt, S. (2003). *Journal de Physique IV*, **104**, 635–638.
